# Dietary Blue Pigments Derived from Genipin, Attenuate Inflammation by Inhibiting LPS-Induced iNOS and COX-2 Expression via the NF-κB Inactivation

**DOI:** 10.1371/journal.pone.0034122

**Published:** 2012-03-30

**Authors:** Qiang-Song Wang, Yaozu Xiang, Yuan-Lu Cui, Ke-Ming Lin, Xin-Fang Zhang

**Affiliations:** 1 Tianjin State Key Laboratory of Modern Chinese Medicine, Tianjin University of Traditional Chinese Medicine, Tianjin, People's Republic of China; 2 Department of Medicine, Imperial College London, Hammersmith Hospital Campus, London, United Kingdom; Universidad Federal de Santa Catarina, Brazil

## Abstract

**Background and Purpose:**

The edible blue pigments produced by gardenia fruits have been used as value-added colorants for foods in East Asia for 20 years. However, the biological activity of the blue pigments derived from genipin has not been reported.

**Methodology/Principal Findings:**

The anti-inflammatory effect of blue pigments was studied in lipopolysaccharide (LPS) stimulated RAW 264.7 macrophage *in vitro*. The secretions of nitric oxide (NO) and prostaglandin E_2_ (PGE_2_) were inhibited in concentration-dependent manner by blue pigments. Real-time reverse-transcription polymerase chain reaction (Real-time RT-PCR) analyses demonstrated that the mRNA expression of inducible nitric oxide synthase (iNOS), cyclooxygenase-2 (COX-2), interleukin (IL)-6, and tumor necrosis factor alpha (TNF-α) was inhibited, moreover, ELISA results showed that the productions of IL-6 and TNF-α were inhibited. Cell-based ELISA revealed the COX-2 protein expression was inhibited. The proteome profiler array showed that 12 cytokines and chemokines involved in the inflammatory process were down-regulated by blue pigments. Blue pigments inhibited the nuclear transcription factor kappa-B (NF-κB) activation induced by LPS, and this was associated with decreasing the DNA-binding activity of p65 and p50. Furthermore, blue pigments suppressed the degradation of inhibitor of κB (IκB) α, Inhibitor of NF-κB Kinase (IKK) α, IKK-β, and phosphorylation of IκB-α. The anti-inflammatory effect of blue pigments *in vivo* was studied in carrageenan-induced paw edema and LPS-injecting ICR mice. Finally, blue pigments significantly inhibited paw swelling and reduced plasma TNF-α and IL-6 production *in vivo*.

**Conclusions and Implications:**

These results suggest that the anti-inflammatory properties of blue pigments might be the results from the inhibition of iNOS, COX-2, IL-6, IL-1β, and TNF-α expression through the down-regulation of NF-κB activation, which will provide strong scientific evidence for the edible blue pigments to be developed as a new health-enhancing nutritional food for the prevention and treatment of inflammatory diseases.

## Introduction

With growing concern on the safety of synthetic dyes, the importance of natural colorants suitable for using in foods has gained increasing attention. Genipin, the aglycon of geniposide, is obtained from the fruit of *Gardenia jasminoides* ELLIS. Genipin itself is colorless but it reacts spontaneously with amino acids to form blue pigments which are used in food industry widely [1]. The edible blue pigments produced by gardenia fruits have been widely used as a blue food colorant in East Asia [2]. Since the blue pigments were used in the food industry, the stability with regard to pH, temperature, and light conditions were also investigated [3], however, very few biological activity studies of the blue pigments are reported.

The inflammation process is crucial to defense against microorganism infection. Key events in the inflammatory process include expression of inflammatory cytokines, chemokines, and other mediators [4]. Macrophages play an important role in inflammatory disease and host defense through the release of factors such as NO, prostaglandin mediators, and cytokines involved in the immune response [5], [6], [7]. LPS is one of the most powerful activators of macrophages known, and macrophages induced by LPS are known to be activated through the production of inflammatory mediators, such as NO and other free radicals, in addition to numerous cytokines, such as TNF-α, IL-1β and IL-6 [8], [9], [10]. NO is a major product which is controlled by nitric oxide synthases (NOS), such as iNOS, eNOS and nNOS [11]. Most importantly, iNOS is highly expressed in macrophages, which leads to organ destruction in some inflammatory and autoimmune diseases [12]. PGE_2_ is also another important mediator which is produced from arachidonic acid metabolites which are catalyzed by COX-2 in inflammatory responses [13].

NF-κB, a nuclear transcription factor, regulates the expression of various genes, including cytokines, iNOS and COX-2, which play critical roles in apoptosis, various autoimmune diseases, and inflammation [14]. NF-κB exists in most cells as homodimeric or heterodimeric complexes of p50 and p65 subunits and remains inactive in the cytoplasm of cells associated with the NF-κB inhibitory protein (I-κB) [15]. NF-κB is activated in response to LPS, which induced NF-κB activation through increasing nuclear p65 protein associated with decreased cytosolic I-κB protein [15]. The resulting free NF-κB is then translocated into the nucleus, where it binds to κB binding sites in the promoter region of target genes, and induces the transcription of pro-inflammatory mediators, including iNOS, COX-2, TNF-α, IL-1β, and others [16], [17], [18]. Because of its ubiquitous role in the pathogenesis of inflammatory gene expression, NF-κB is a current target for treating various diseases [19], [20].

The macrophage cell line (RAW 264.7) used in experiments has been established as a suitable model to investigate compounds interfering with LPS-inducible inflammatory cascades *in vitro* [21], [22], [23], [24], [25]. In this study, the anti-inflammatory effects of the blue pigments on the generation of several chemokines, cytokines and enzymes involved in the inflammatory process, such as NO, PGE_2_, TNF-α, IL-6, IL-1β, iNOS and COX-2 in LPS-induced RAW 264.7cells were investigated. We also investigated whether the blue pigments influence the LPS induced DNA binding activity of NF-κB and the protein level of its subunit, p65 and p50.

## Methods

### Ethics statement

The approved ID of the mice experiments is TCM-2009-037-E05.

This work was supported by the National Natural Science Foundation of China (30973967, 81173469), which has been inspected by the Animal Ethics Committee of Tianjin University of Traditional Chinese Medicine. The ICR mice used in our work were obtained from Huafukang Bio-technology Co. Ltd. (SCXK 2009-0004, Beijing, China).

### Reagents

Dulbecco's modified Eagle's medium-high glucose (DMEM), 2-(4, 5-dimethylthiazol-2-yl)-2, 5-diphenyltetrazolium bromide (MTT), Lipopolysaccharides from Escherichia coli 0111:B4 (LPS) were purchased from Sigma-Aldrich Co. (USA). Prostaglandin E_2_ Express EIA Monoclonal Kit was obtained from Cayman Chemical (USA). IL-6 Mouse ELISA Kit and TNF-α Mouse ELISA Kit were obtained from Invitrogen (USA). 4-amino-5-methylamino- 2′, 7′-difluorofluorescein diacetate (DAF-FM diacetate) was purchased from Invitrogen (USA). BCA Protein Assay Kit was obtained from Pierce (USA). Mouse Cytokine Array Panel A Array kit was purchased from R&D Systems, Inc. (USA). Mammalian Cell Lysis Kit and UNIQ-10 column Trizol total RNA extraction kit were bought from Sangon Biological Engineering Technology & Services Co., Ltd. (Shanghai, China). Improm-II Reverse Transcription System was purchased from Promega Corporation (USA). FastStart Universal SYBR Green Master (ROX) kit was purchased from Roche (Germany). Mouse Anti-COX-2 Monoclonal Antibody was from BD Pharmingen (USA) and Goat Anti-Mouse IgG Peroxidase Conjugate was from Calbiochem (Germany). Nuclear Extract Kit was purchased from Active Motif (Japan). Universal EZ-TFA Transcription Factor Assay and NF-κB Family EZ-TFA Transcription Factor Assay kits were purchased from Millipore (USA). P-IκB-α, IκB-α, IKK-α, IKK-β monoclonal antibodies and peroxidase-conjugated secondary antibody were purchased from Cell Singaling Technology (USA), and β-actin monoclonal antibody was purchased from Sigma-Aldrich Co. (USA). NF-κB inhibitor BAY 11-7082 was purchased from Beyotime Institute of Biotechnology (China). Carrageenan was purchased from Sigma-Aldrich Co. (USA) and Dexamethasone was purchased from Shanghai General Pharmaceutical Co., Ltd.(Shanghai, China).

### Preparation of the blue pigments

**Figure 1:**
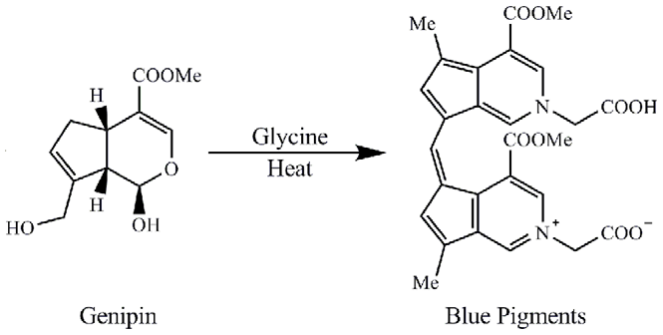
The proposed formation structure of blue pigments from genipin with glycine.

Genipin was purchased from Wako (Osaka, Japan). Dietary blue pigments derived from genipin were prepared according to the methods described earlier [26]. Briefly, 8.8 mmol of genipin and amino acids (glycine, 8.8 mmol) were added respectively into 400 mL of 100 mM phosphate buffer (pH 7.0) at 80°C and stirred for 4 h. The blue pigments were passed through Diaion HP-20 resin and ODS columns chromatography and the fractions at 595 nm were collected. The blue pigments were cold-sterilized using a 0.22-µm pore size membrane filter (Millipore, USA) and stored in the refrigerator for further use. The proposed formation structure of blue pigments from genipin with glycine were shown in Figure 1 [26]. The concentrations of blue pigments were calculated by the molar mass of genipin.

### Cells and cell culture

RAW 264.7 murine macrophages cell line was obtained from Cell Culture Center of Chinese Academy of Medical Sciences (Beijing, China). RAW 264.7 cells were maintained in DMEM supplemented with 10% heat inactivated fetal bovine plasma (HI-FBS), 100 U/mL penicillin and 100 µg/mL streptomycin at 37°C in a humidified incubator containing 5% CO_2_. For the determination of cell viability, nitrite concentration as an index for NO synthesis, PGE_2_ concentrations, as well as different acute phase proteins, cytokines, and chemokines in culture medium, the cells were plated at 5×10^5^ cells/well in 96-well plates and treated with various concentrations of blue pigments in the presence of 0.2 µg/mL LPS for 18–24 h as indicated. Moreover, for the determination of protein levels of COX-2, cells were treated with various concentrations of blue pigments and in the presence of 0.2 µg/mL LPS for 24 h. For real-time RT-PCR, the cells were pre-incubated with various concentrations of blue pigments for 2 h and were then treated with 0.2 µg/mL LPS for an additional 6 h. The blue pigments at various concentrations dissolved in Phosphate buffered saline (PBS, pH 7.4) were added together with LPS. Cells were treated with PBS as vehicle control.

### Cell viability assay (MTT assay)

RAW 264.7 cells were treated with various concentrations of blue pigments (12.5, 25, 50, 100 and 200 µM) for 24 h. Then MTT (stock solution of 5 mg/mL) was added to a final concentration of 0.5 mg/mL, and the cells were incubated for an additional 4 h at 37°C and 5% CO_2_. The medium was removed and the formazan precipitate was solubilized in 100 µL DMSO, and the absorbance was measured at 570 nm on a multifunctional microplate reader (FlexStation 3, Molecular Devices, USA).

### Nitrite assay, detection of intracellular NO production, PGE_2_ and cytokines

RAW 264.7 cells were treated with various concentrations of blue pigments (12.5, 25, 50, 100 µM) in the presence of 0.2 µg/mL LPS. 18 hours later, the medium was collected. The nitrite accumulated in culture medium was measured as an indicator of NO production based on a diazotization reaction using Griess reagent system (Promega, USA). Nitrite concentration was determined by a standard curve prepared with sodium nitrite dissolved in DMEM without phenol red supplemented with 5% HI-FBS. Intracellular NO production was evaluated by confocal laser scanning microscopy of a fluorescent NO derivative. LPS stimulated and blue pigments-treated RAW 264.7 cells were seeded in 4-well chamber slides (Millicell EZ Slide, Millipore, USA) and incubated for 1 h at 37°C with 10 µM DAF-FM diacetate. Digitized images were generated with a Zeiss LSM 710 confocal laser scanning microscope (Carl Zeiss Microimaging, Jena, Germany) with an excitation wavelength of 495 nm and an emission wavelength of 515 nm. PGE_2_ production was measured with the Prostaglandin E_2_ Express EIA Monoclonal Kit (Cayman Chemical, USA). IL-6 and TNF-α secreted in the culture medium were quantified by ELISA kits (Invitrogen, USA) according to the manufacturer's instructions. Samples were assayed after a 10-fold dilution in the medium (DMEM without phenol red supplemented with 5% HI-FBS).

### Cell-based ELISA for COX-2 protein expression in RAW 264.7 cells

RAW 264.7 cells were seeded in 96-well plates treated with various concentrations of blue pigments (12.5, 25, 50, 100 µM) in the presence of 0.2 µg/mL LPS. The expression of COX-2 was determined by a slightly modified original protocol for the cell-based ELISA [27], [28]. After cultivation, cells were fixed with 4% paraformaldehyde in phosphate-buffered saline (PBS, pH 7.4) for 20 min at room temperature and washed three times with PBS containing 0.1% Triton X-100 (PBS/T). Endogenous peroxidase was quenched with 0.6% H_2_O_2_ in PBS/T for 20 min, and cells were washed three times in PBS/T. Following blocking with 10% FCS in PBS/T for 1 h, cells were incubated for 2 h at 37°C with the primary antibody in PBS/T containing 1% BSA. After washing the cells four times with PBS/T for 5 min, the plate was incubated for 1 h at room temperature with secondary antibody in PBS/T containing 1% BSA. Subsequently, cells were washed and incubated with TMB substrate solution (Invitrogen, USA) for 30 min at room temperature in the dark. The reaction was stopped with 50 µL of 2 N H_2_SO_4_, and the absorbance at 450 nm was determined on a multifunctional microplate reader. The data were corrected for differences in cell number by staining the cells with Janus Green B after the cell-based ELISA procedure [29].

### Cytokine protein array analyses

RAW 264.7 cells were seeded in 6-well plates treated with blue pigments (100 µM) in the presence of 0.2 µg/mL LPS for 18 h. Cells were collected by centrifugation and washed once with PBS. The washed cell pellets were resuspended in extraction lysis buffer (Sangon, China) and incubated with 20 min at 4°C. The protein concentration was determined using the Pierce protein assay reagent according to the manufacture's instruction. Screening for different acute phase proteins, cytokines, and chemokines in cell lysates were performed with a Proteome profiler array (Mouse Cytokine Array Panel A Array kit) from R&D Systems [30], [31], [32]. The array allows to detect the following proteins: BLC, C5a, G-CSF, GM-CSF, I-309, Eotaxin, sICAM-1, IFN-γ, IL-1α, IL- 1β, IL-1ra, IL-2, IL-3, IL-4, IL-5, IL-6, IL-7, IL-10, IL-12p70, IL-13, IL-16, IL-17, IL-23, IL-27, IP-10, I-TAC, KC, M-CSF, JE, MCP-5, MIG, MIP- 1α, MIP-1β, MIP-2, RANTES, SDF-1, TARC, TIMP-1, TNF-α and TREM-1. Horseradish peroxidase substrate (Millipore Corporation, USA) was used to detect protein expression and data were captured by exposure to Kodak BioMax Light films. Films were scanned into a computer and densitometry was performed using the Image-Pro Plus version 6.0 (Media Cybernetics, Silver Spring, MD, USA).

### Real-time RT-PCR for detecting mRNA expression of TNF-α, COX-2, iNOS, IL-6

Total RNA was isolated using Sangon UNIQ-10 column Trizol total RNA extraction kit according to the instructions of the manufacturer. RNA (1 µg) was reversely transcribed using ImProm-II Reverse Transcription System cDNA synthesis kit. The real-time RT-PCR oligonucleotide primers used for mouse iNOS, COX-2, IL-6, TNF-α and β-actin are shown in Table 1. The reactions were setup in duplicates in 25 µL total volumes with 1 µL of each primer (0.3 µM final concentrations), 12.5 µL of FastStart Universal SYBR Green Master (ROX) (Roche), and 1 µL of template. The PCR cycle was as follows: 95°C for 10 min, 40 cycles of 95°C for 15 s, 60°C for 1 min, and a melt curve analysis was performed at the end of each experiment to verify that a single product per primer pair was amplified. The amplification and analysis were performed using an ABI Prism 7500 Real-Time PCR System. Samples were compared using the relative CT method. The fold increase or decrease was determined relative to a blank control after normalized to a housekeeping gene using 2^−ΔΔC^T [33], [34].

**Table 1:** The real-time RT-PCR oligonucleotide primers.

Gene	primer	sequence (5′-3′)	PCR product (bp)
β-actin	forward	AGAGGGAAATCGTGCGTGAC	138
(NM_007393.3)	reverse	CAATAGTGATGACCTGGCCGT	
iNOS	forward	GGCAGCCTGTGAGACCTTTG	72
(NM_010927.3)	reverse	GCATTGGAAGTGAAGCGTTTC	
COX-2	forward	TGAGTACCGCAAACGCTTCTC	151
(NM_011198.3)	reverse	TGGACGAGGTTTTTCCACCAG	
IL-6	forward	TCCAGTTGCCTTCTTGGGAC	140
(NM_031168.1)	reverse	GTGTAATTAAGCCTCCGACTTG	
TNF-α	forward	TTCTGTCTACTGAACTTCGGGGTGATCGGTCC	354
(NM_013693.2)	reverse	GTATGAGATAGCAAATCGGCTGACGGTGTGGG	

### NF-κB activity assay

RAW 264.7 macrophages cells were plated in 60-mm dishes (2×10^6^ cells/dish). The cells were treated with various concentrations (25, 50, 100 µM) of blue pigments for 2 h, stimulated with LPS for 30 min, washed three times with cold PBS. Cells were collected by centrifugation and washed once with PBS. The washed cell pellets were resuspended in extraction lysis buffer (Sangon, China) (50 mM HEPES pH 7.0, 250 mM NaCl, 5 mM EDTA, 0.1% Nonidet P-40, 1 mM PMSF, 0.5 mM DTT, 5 mM NaF and 0.5 mM sodium orthovanadate) containing 5 µg/ml of leupeptin and aprotinin, respectively, and incubated with 20 min at 4°C. Cell debris was removed by centrifugation, and supernatants were rapidly frozen. The protein was detected by BCA method (Pierce, USA). Furthermore, nuclear extracts were prepared with the manufacture's instruction (Active Motif, Japan). Briefly, Cell pellets were resuspended in hypotonic buffer (10 mM HEPES, pH 7.9, 1.5 mM MgCl_2_, 10 mM KCl, 0.2 mM PMSF, 0.5 mM DTT, 10 mg/mL aprotinin) and incubated on ice for 15 min. They were then lysed by adding 0.1% Nonidet P-40 and vortexing vigorously for 10 s. Nuclei were pelleted by centrifugation at 12,000 g for 10 min at 4°C and resuspended in high salt buffer (20 mM HEPES, pH 7.9, 25% glycerol, 400 mM KCl, 1.5 mM MgCl_2_, 0.2 mM EDTA, 0.5 mM DTT, 1 mM NaF, 1 mM sodium orthovanadate). Cell debris was removed by centrifugation, and supernatants were rapidly frozen. The DNA-binding activity of NF-κB p50 and p65 was detected with universal EZ-TFA transcription factor assay kit (Millipore, USA) as the manufacturer's instructions. The expressions of IKK α, IKK β, IκB-α, P- IκB-α were analyzed by western blot. Of cellular protein, 40 mg from treated and untreated cell extracts were electro-blotted onto a polyvinylidene difluoride (PVDF) membrane following separation on a 10% SDS-polyacrylamide gel electrophoresis. The immunoblot was incubated for 2 hours with blocking solution (5% skim milk) at room temperature, and then incubated overnight with a 1∶1000 dilution of anti- IKK α, anti- IKK β, anti-IκB-α, P-IκB-α, β-actin antibody at 4°C (Cell Singaling Technology, USA). Blots were washed five times with Tween 20/Tris-buffered saline (TTBS) and then incubated with a 1∶ 1000 dilution of horseradish peroxidase-conjugated secondary antibody (Cell Singaling Technology, USA) for 1 h at room temperature. Blots were again washed five times with TTBS and then developed by Horseradish peroxidase substrate (Millipore Corporation, USA) and data were captured by exposure to Kodak BioMax Light films.

### Animals

Male ICR strain of mice (4–5 weeks of age), purchased from Beijing Huafukang Bio-technology Co. Ltd., were group-housed under controlled light (12-h/12-h light–dark cycle; lights on at 07:00 a.m.) in the Laboratory Animal Center of Tianjin University of Traditional Chinese Medicine. Ambient temperature and relative humidity were maintained at 24±1°C and 55±5%, respectively. All the animals had free access to water and food in a home cage.

### Carrageenan-induced paw edema test in mice

Paw edema was induced by subcutaneous injection of 50 µL of 1% carrageenan into the right hind paw of mice as previously described [35]. The mice were divided into 5 groups: vehicle control group, 1% carrageenan+0.9% saline; 1% carrageenan+Dexamethasone (10 mg/kg); or 1% carrageenan+blue pigments (30, 60, 120 mg/kg) (n = 10 for each group). Groups were pretreated intraperitoneally (i.p.) with 200 µL of 0.9% saline, Dexamethasone or Genipin blue (30, 60, 120 mg/kg) for 30 minutes. 50 µL of 1% carrageenan was then administered in 200 µL i.p for every group. The paw thickness was measured using a 7140 Plethysmometer (UGO BASILE, Italy) before and every hour after edema induction for 4 h. The percent increase of paw thickness was calculated based on the volume difference between the paw with and without carrageenan injection.

### Plasma concentrations of TNF-α and IL-6 in LPS-stimulated ICR mice

The mice were divided into 6 groups: negative control group, injected with 0.9% saline; LPS+0.9% saline; LPS+Dexamethasone (10 mg/kg); or LPS+blue blue pigments (30, 60, 120 mg/kg) (n = 8 for each group). Groups were pretreated intraperitoneally (i.p.) with 200 µL of 0.9% saline, Dexamethasone or genipin blue for 30 minutes. Lipopolysaccharide (1 mg/kg) was then administered in 200 µL i.p for 5 treatment groups, and 0.9% saline was administered i.p. for the negative control group. Blood was withdrawn from the animals under ether anesthesia ninety minutes later [36]. The centrifuge tube was heparinized and blood from mice was centrifuged at 2000 rpm for 15 minutes, and the plasma was collected and stored at −80°C until analysis. Plasma levels of TNF-α and IL-6 were determined using Mouse ELISA kit (Invitrogen, USA).

### Statistical analysis

One-way ANOVA or *t*-test was used for determining the statistically significant differences between the values of various experimental groups. Data were expressed as means ± SD and a *P* value<0.05 was considered statistically significant.

## Results

### Effect of blue pigments on RAW 264.7 cells viability

The cytotoxicity of blue pigments on RAW 264.7 cells was measured with MTT assay. Cell viability was not significantly altered by blue pigments at up to 200 µM. These results suggest that concentrations of blue pigments below 200 µM are not toxic to RAW 264.7 cells. Therefore, for all experiments, cells were treated with blue pigments in the concentration range of 12.5–100 µM. The MTT assay results of blue pigments on RAW 264.7 cells were shown in Figure S1.

### Effect of blue pigments on LPS-induced NO production

**Figure 2:**
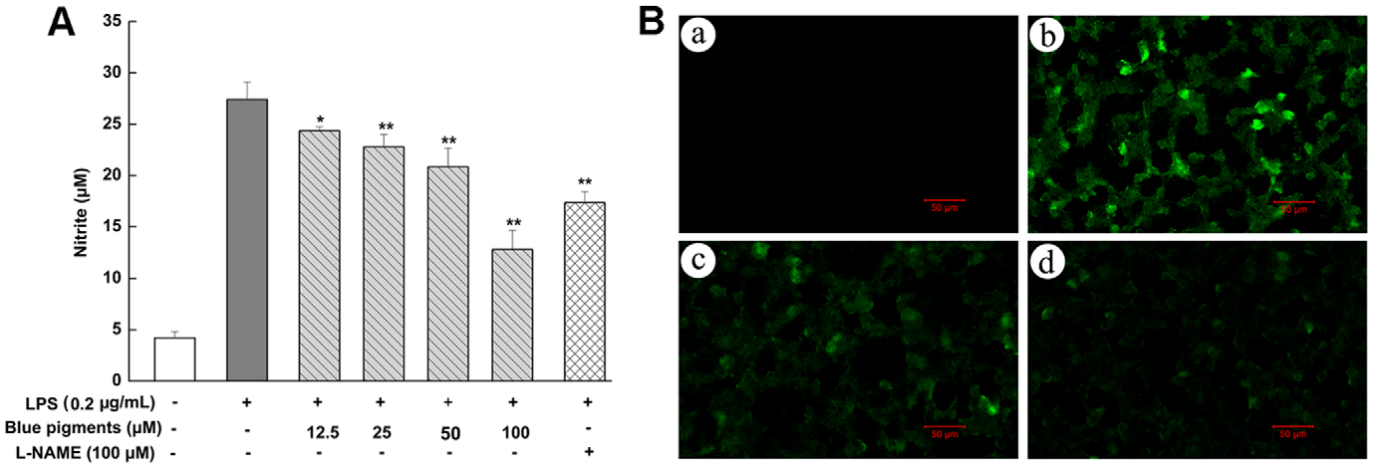
The effect of blue pigments on LPS-induced NO in RAW 264.7 cells. (A) RAW 264.7 cells were incubated with the indicated concentrations of blue pigments and 0.2 µg/mL LPS for 18 h. The NO content of culture medium was analyzed by Griess reagent system. Data represent means ±S.D. values from three independent experiments. * *P*<0.05, ** *P*<0.01 (n = 6) compared with LPS treated cells alone. (B) RAW 264.7 cells were incubated with the indicated concentrations of blue pigments and 0.2 µg/mL LPS for 18 h. Intracellular NO production was evaluated with DAF-FM diacetate by confocal laser scanning microscopy: (a) control (cells alone); (b) cells stimulated with LPS; (c) 100 µM blue pigments was added under the condition of part (b); (d) 100 µM L-NAME was added under the condition of part (b).

The effect of blue pigments on NO in LPS-induced RAW 264.7 cells was tested to investigate the anti-inflammatory effects. Concentrations of nitrite accumulated in the culture medium were estimated by Griess reagent as an index for NO. RAW 264.7 cells were pretreated with different concentrations (12.5, 25, 50, 100 µM) of blue pigments, which were found to significantly inhibit LPS-induced NO production in a concentration-dependent manner (*P*<0.01). The NO inhibitor, L-NAME (100 µM), as a positive control, also inhibited the production of NO in activated RAW 264.7 cells (Figure 2A). Furthermore, confocal laser scanning microscopy also showed blue pigments to be a stronger inhibitor of intracellular NO production than that in single LPS stimulation (Figure 2B).

### Effect of blue pigments on LPS-induced cytokines TNF-α and IL-6

**Figure 3:**
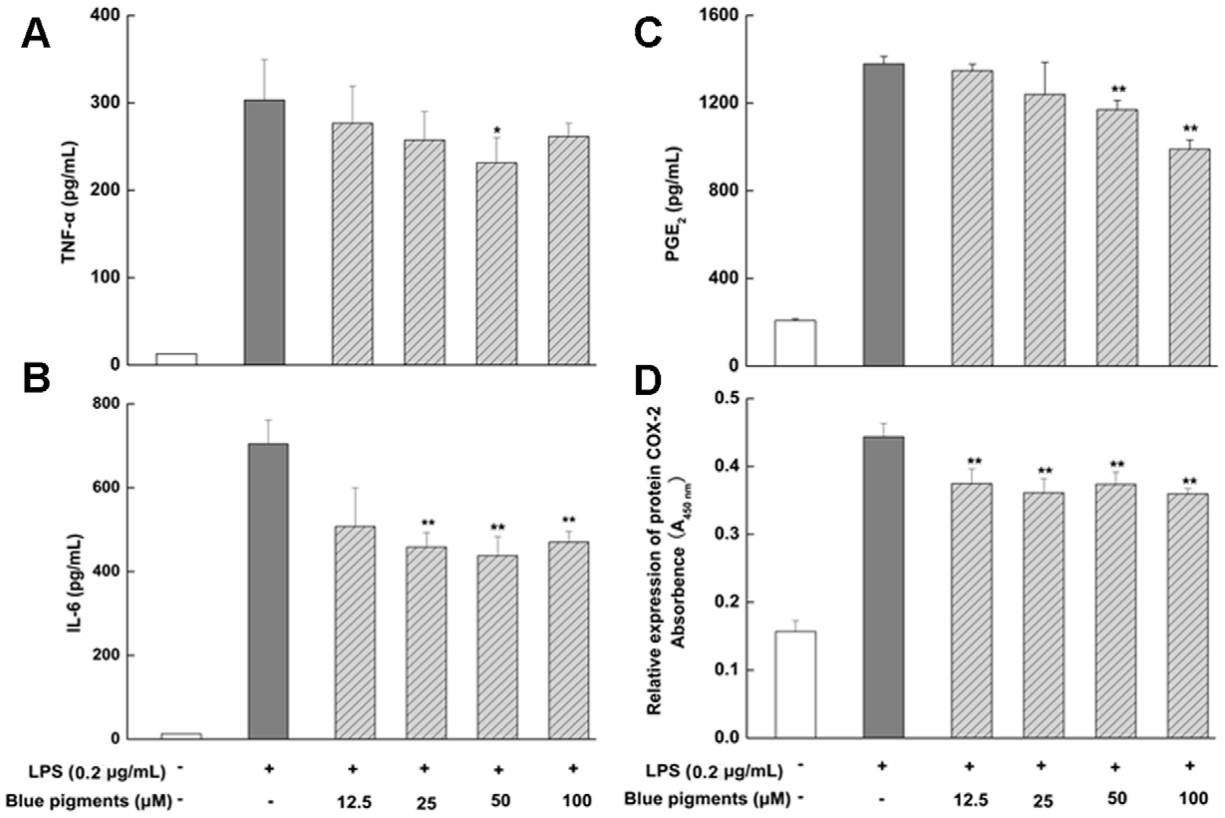
Effect of blue pigments on LPS-induced TNF-α, IL-6, PGE_2_ production and COX-2 protein expression. RAW 264.7 cells were incubated with the indicated concentrations of blue pigments and 0.2 µg/mL LPS for 18 h or 24 h. TNF-α (A), IL-6 (B), PGE_2_ (C) in the culture medium were analyzed by ELISA, and the COX-2 protein expression (D) was analyzed by cell-based ELISA. Data represent means ±S.D. values from three independent experiments. * *P*<0.05, ** *P*<0.01 (n = 6) compared with LPS treated cells alone.

TNF-α, IL-6 are known to be pro-inflammatory cytokines that posses a multitude of biological activities linked to the immune-pathology of acute or chronic inflammatory diseases. After treatment with blue pigments and activated with LPS (0.2 µg/mL), the secretion of IL-6 and TNF-α were detected by ELISA. As shown in Figure 3A–B, pretreatment of RAW 264.7 cells with blue pigments (25, 50, 100 µM) significantly reduced IL-6 production (*P*<0.01), whereas TNF-α production were only inhibited slightly by 50 µM blue pigments (*P*<0.05).

### PGE_2_ production and Protein expression of COX-2 on blue pigments

LPS-induced PGE_2_ production was detected by ELISA. As shown in Figure 3C, blue pigments inhibited the production of PGE_2_. Blue pigments (50, 100 µM) had a markedly higher inhibitory effects (*P*<0.01). In order to investigate whether the inhibition of PGE_2_ production was due to a decreased protein expression of COX-2, the effect of blue pigments on COX-2 protein expression was studied by cell-based ELISA. As shown in Figure 3D, LPS treatment significantly increased COX-2 protein expression levels, whereas COX-2 protein expression was suppressed the induction of blue pigments (*P*<0.01 or *P*<0.05).

### Effect of blue pigments on multiple cytokines

**Figure 4:**
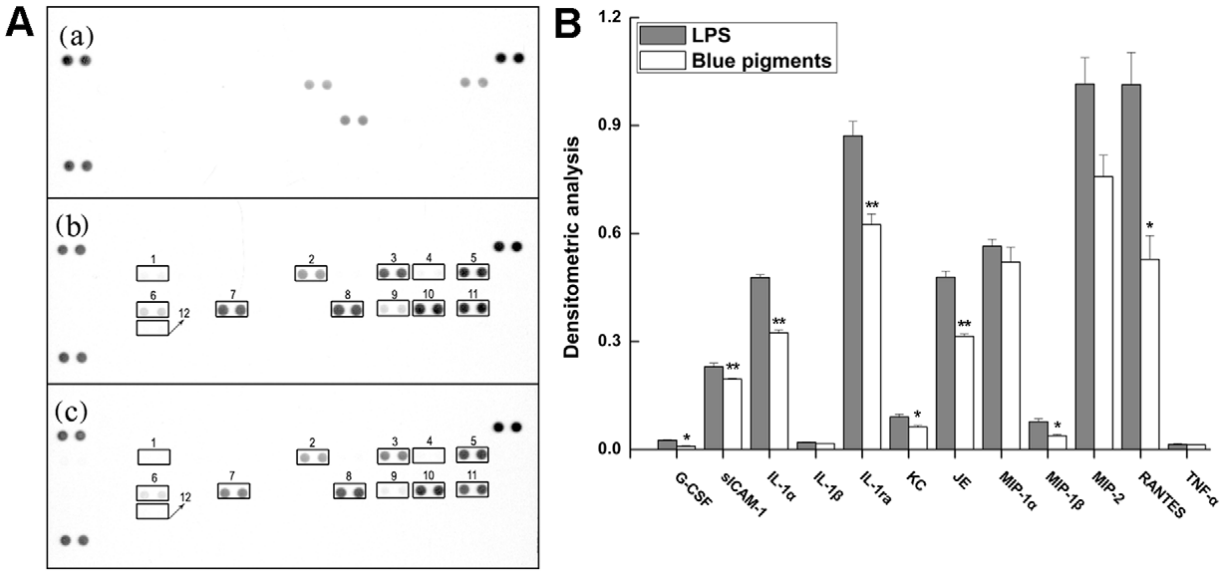
Effect of blue pigments on LPS-stimulated multiple cytokines produced in RAW 264.7 cells. RAW 264.7 cells were incubated with the concentrations of blue pigments and 0.2 µg/mL LPS for 18 h. (A) The R&D Systems Mouse Cytokine Antibody Proteome Profiler Array system was used to screen for activation of different acute phase proteins, cytokines, and chemokines involved in the inflammatory process in RAW264.7 cells. (a) Control; (b) RAW 264.7 cells were induced by LPS (0.2 µg/mL); (c) RAW 264.7 cells were treated with blue pigments in the presence of LPS (0.2 µg/mL). The cytokines in cell lysates were analyzed by Proteome profiler array. Presented numbers on membranes mark the following targets: “1” G-CSF; “2” sICAM-1; “3” IL-1α; “4” IL-1β; “5” IL-1ra; “6” KC; “7” JE; “8” MIP-1α; “9” MIP-1β; “10” MIP-2; “11” RANTES; “12” TNF-α. (B) Quantification of cytokines optical density. Measurement was obtained with the Image-Pro Plus version 6.0.

In Inflammatory, multiple cytokines secreted in macrophages were activated significantly. After stimulating with 0.2 µg/mL LPS, multiple cytokines such as, G-CSF, sICAM-1, IL-1α, IL-1β, IL-1ra, KC, JE, MIP-1α, MIP-1β, MIP-2, RANTES and TNF-α were up-regulated. However, the expressions of G-CSF, sICAM-1, IL-1α, IL-1ra, KC, JE, MIP-1β, RANTES could be significantly down-regulated by the blue pigments (Figure 4B *P*<0.01 or *P*<0.05, the optical density data are presented in Table S1), however, the expressions of IL-1β, MIP-2, TNF-α were inhibited by blue pigments, but no significant (*P*>0.05). The blue pigments could modulate the synthesis of several cytokines which were involved in the inflammatory process.

### Effect of blue pigments on LPS-induced mRNA expression of TNF-α, IL-6, iNOS and COX-2

**Figure 5:**
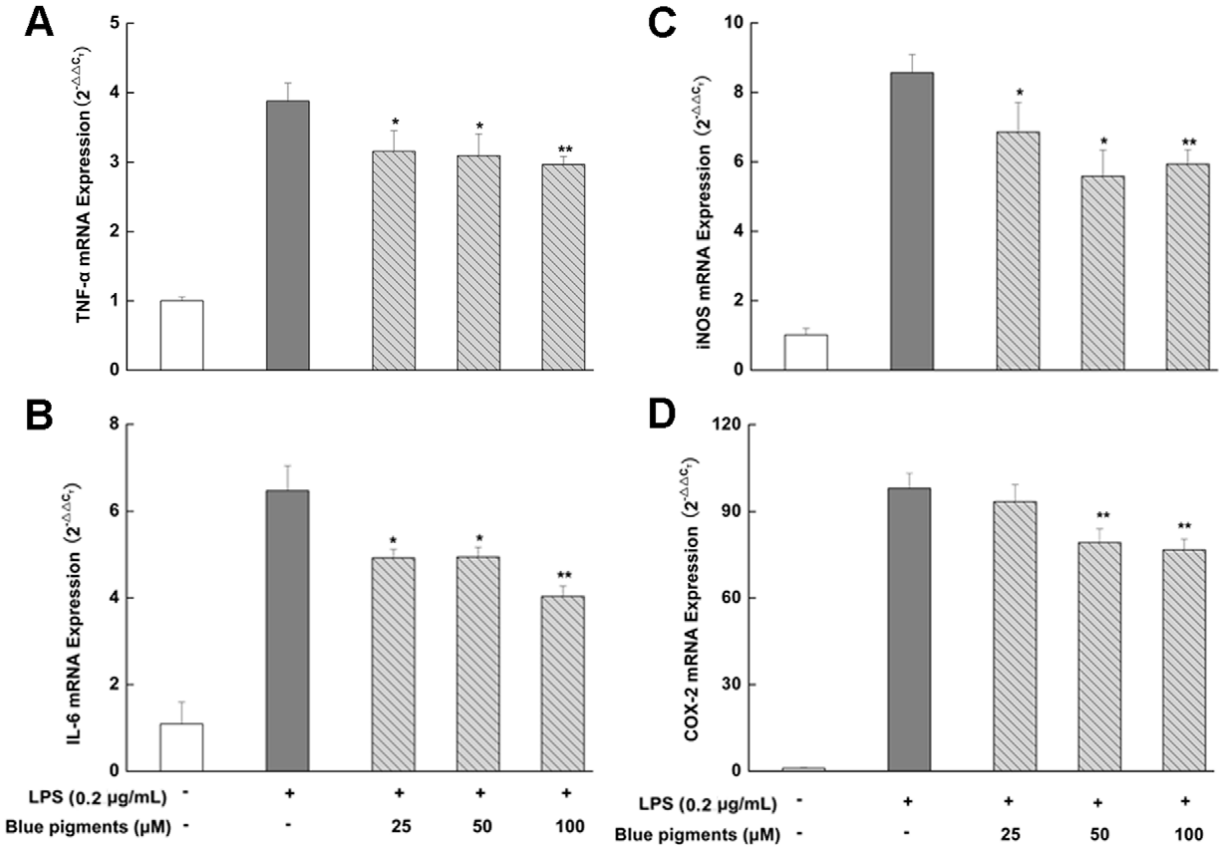
Effect of blue pigments on LPS-stimulated mRNA expression of TNF-α, IL-6, iNOS and COX-2. RAW 264.7 cells were pre-incubated with indicated concentrations of blue pigments for 2 h and were then treated with 0.2 µg/mL LPS for an additional 6 h. The mRNA expression of TNF-α (A), IL-6 (B), iNOS (C) and COX-2 (D) was analyzed by real-time RT-PCR. Data represent means ± S.D. values from three independent experiments. * *P*<0.05, ** *P*<0.01 (n = 6) compared with LPS treated cells alone.

Since blue pigments was found to most potently inhibit the pro-inflammatory mediators, e.g., NO, PEG_2_, TNF-α, IL-6 in supernatants, we investigated the effects of blue pigments on LPS-induced iNOS, COX-2, TNF-α, IL-6 gene expression using real-time RT-PCR. The inhibitions of blue pigments on LPS-induced mRNA expression of TNF-α, IL-6, iNOS, COX-2, were observed in Figure 5A–D. The results showed that the effect of blue pigments on mRNA expression of TNF-α, IL-6, iNOS, COX-2 was coincidence with the secretion of TNF-α, IL-6, NO, PEG_2_ that in culture medium. Moreover, the mRNA expression of COX-2 was significantly inhibited by the blue pigments, which was coincidence with the expression of COX-2 protein.

### Effects of blue pigments on NF-κB activity

**Figure 6:**
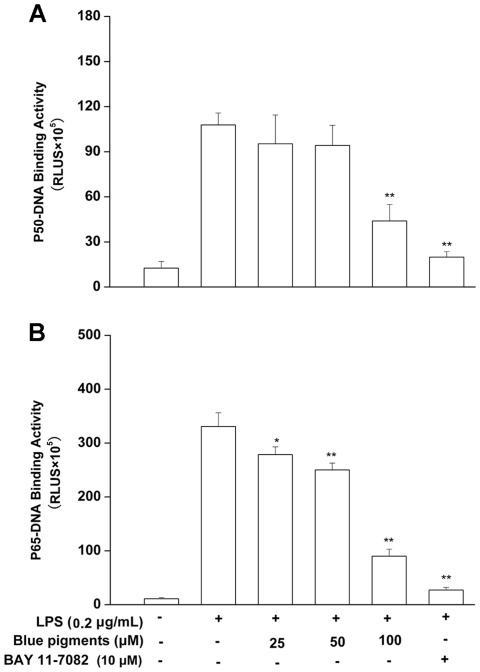
Effects of blue pigments on LPS-induced NF-κB translocation. RAW 264.7 cells were pre-incubated with indicated concentrations of blue pigments for 2 h and stimulated with LPS (0.2 µg/mL) for 30 min. The DNA-binding activity of NF-κB p50 (A) and p65 (B) was detected with universal EZ-TFA transcription factor assay (Millipore, USA). NF-κB inhibitor BAY 11-7082 (10 µM) was used as positive control. Data represent means ± S.D. values. * *P*<0.05, ** *P*<0.01 (n = 5) compared with LPS treated cells alone.

**Figure 7:**
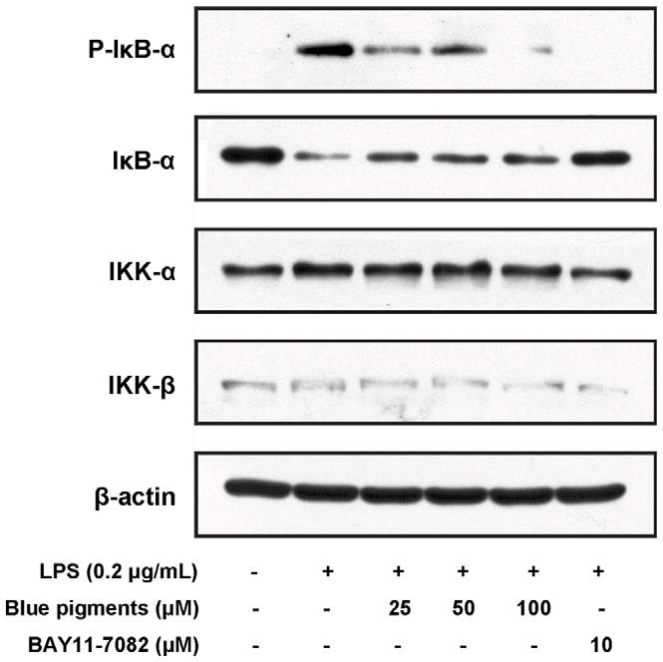
Effects of blue pigments on the degradation of IκB-α, IKK-α, IKK-β and phosphorylation of IκB-α. RAW 264.7 cells were pre-incubated with indicated concentrations of blue pigments for 2 h and stimulated with LPS (0.2 µg/mL) for 30 min. Total cellular proteins were then prepared and Western blotted for IκB-α, IKK-α, IKK-β and p-IκB-α using specific IκB-α, IKK-α, IKK-β and p-IκB-α antibodies. The β-actin protein was used as internal controls. NF-κB inhibitor of BAY 11-7082 (10 µM) was used as positive control.

As the activation of NF-κB is critically required for the activations of iNOS, COX-2, TNF-α, PEG_2_ and IL-6 by LPS, we determined the DNA-binding activity of NF-κB subunits p50 and p65 using the Universal EZ-TFA transcription factor colorimetric assay, which instead of the DNA-binding principle of the electrophoretic mobility shift assay with the 96-well format of an enzymelinked immunosorbent assay. Accordingly, a NF-κB DNA binding assay was carried out using nuclear extracts from RAW 264.7 cells stimulated with LPS in the presence or absence of blue pigments. Treatment of RAW 264.7 cells with LPS (0.2 µg/mL) was found to increase the expression of NF-κB subunits p50 and p65, however, the expressions of p50 (Figure 6A) and p65 (Figure 6B) pretreated these cells with blue pigments prior to LPS were reduced in a concentration-dependent manner when compared with the single LPS stimulation group (*P*<0.01). Taken together, the above findings showed that blue pigments suppressed iNOS, COX-2, TNF-α, PEG_2_ and IL-6 expression at least in part via an NF-κB-dependent mechanism. We also explored whether blue pigments inhibited the LPS-stimulated degradation of IκB-α in RAW 264.7 cells by Western blotting with anti-IκB-α antibody. Figure 7 shows that LPS-induced IκB-α degradation was significantly blocked by blue pigments pretreatment. Furthermore, to determine whether this IκB-α degradation was related to IκB-α phosphorylation, we examined the effect of blue pigments on the LPS-induced p-IκB-α by Western blotting, and found that blue pigments also significantly reduced LPS-induced IκB-α phosphorylation. The β-actin protein was used as internal controls. Since IKK-α and -β are upstream kinases of IκB in the NF-κB signal pathway [17], we examined the effects of blue pigments on LPS induced IKK-α, -β activation by immunoblotting using IKK-α, -β antibodies. Blue pigments (100 µM) inhibited the expression of IKK-α and IKK-β. The β-actin protein was used as internal control. (The western blot bands were quantified by densitometry and the data are presented in Figure S2).

### Effects of blue pigments on carrageenan-induced paw edema in mice

**Figure 8:**
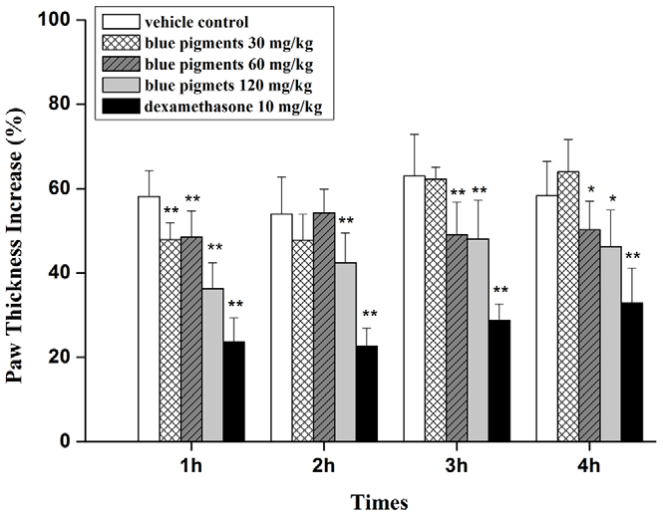
Effects of blue pigments on carrageenan-induced paw edema in mice. Blue pigments (30, 60, 120 mg/kg), dexamethasone (10 mg/kg) or with 0.9% saline were administered 30 min before carrageenan injection into mice for alleviation of acute inflammation. Paw thickness was measured using Plethysmometer before and every hour after edema induction for 4 h. The percent increase of paw thickness was calculated based on the volume difference between the paw with and without carrageenan injection. Data represent means ± S.D. values. * *P*<0.05, ** *P*<0.01 (n = 10) indicate significant differences from vehicle control.

The anti-inflammatory effect of blue pigments was examined using the carrageenan-induced paw edema model. As shown in Figure 8, treatment with blue pigments (60, 120 mg/kg) showed significant inhibitory effects on paw swelling, compared with the vehicle control group. Maximal edema inhibition was observed at 1 h after edema induction. Notably, treatment with blue pigments (120 mg/kg) reduced edema by 21.9% at 1 h, whereas the positive control, Dexamethasone (10 mg/kg) decreased the edema rate by 34.5% at 1 h.

### Effects of blue pigments on plasma concentrations of TNF-α and IL-6 in LPS-stimulated ICR mice

**Figure 9:**
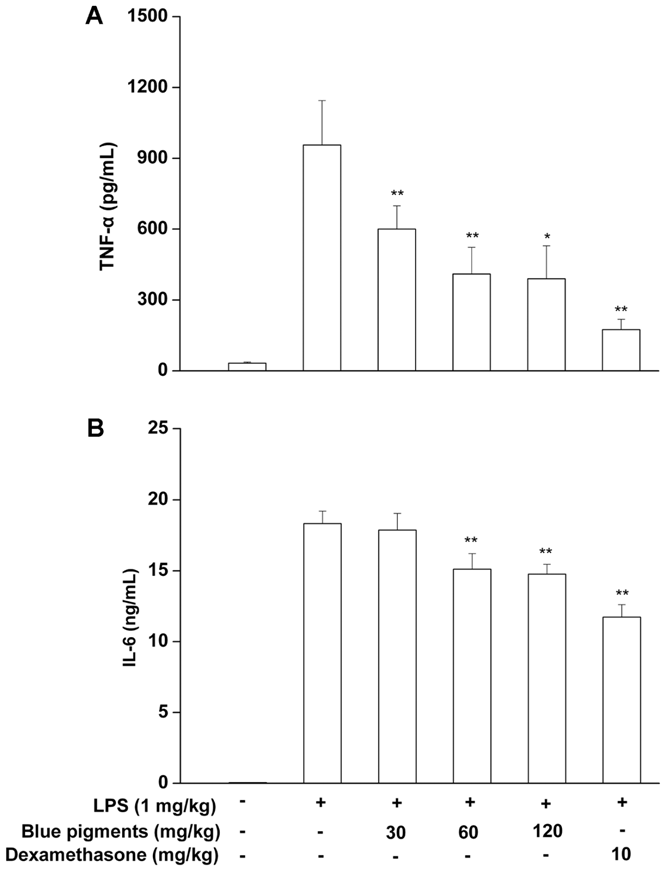
Effects of blue pigments on plasma concentrations of TNF-α and IL-6 in LPS-stimulated ICR mice. Blue pigments (30, 60, 120 mg/kg), dexamethasone (10 mg/kg) or 0.9% saline were pretreated for 30 min. The mice were then either injected with 1 mg/kg lipopolysaccharide (LPS) or PBS for 90 min. Plasma TNF-α (A) and IL-6 (B) were quantified using ELISA. Data represent means ±S.D. values. * *P*<0.05, ** *P*<0.01 (n = 8) compared with LPS treated alone.

Injecting mice with LPS lead to increase in plasma TNF-α and IL-6 levels compared to untreated mice. Blue pigments significantly reduced the plasma TNF-α and IL-6 levels in a dose-dependent manner in the LPS-stimulated animals (Figure 9). Blue pigments reduced the plasma TNF-α and IL-6 levels by 59.2% and 19.5% in the LPS-stimulated animals. Pretreatment with the anti-inflammatory steroid dexamethasone caused 81.7% and 36.1% reduction in plasma TNF-α and IL-6 in LPS-stimulated mice.

## Discussion

The pharmacological studies showed that genipin had exhibited neuroprotective effect [37], anti-inflammatory effect [38] and suppression of fas-induced lethal liver apoptosis *in vitro* [39].

Because it was a naturally occurring crosslinking reagent with low cytotoxicity, genipin has recently been investigated as a cross-linking regent in many biological applications. Recent explorations into using of genipin cross-linked gelatin as a wound-dressing membrane [40], bioadhesives [41], bone substitute [42] and heparin immobilization [43], have shown its potential as a new and safe cross-linking agent, which had low cytotoxicity and inflammatory reaction, satisfactory biocompatibility and stabile mechanical properties than glutaraldehyde. Genipin was also being investigated in the field of forensic science as a fingerprint reagent to develop latent fingerprints on paper products [44]. Furthermore, genipin could react with amino acid to form the stable blue pigments, and the blue pigments have been approved by Ministry of Public Health of the People's Republic of China used as value-added colorants for foods in 1990 [45]. The biological activity of genipin have been reported widely, however, the biological activity studies of blue pigments have been not reported. Dietary lutein, one of natural colorants for foods, has been shown the potential anti-inflammatory effect *in vitro* [46], and the present study was elucidating the molecular mechanisms underlying the anti-inflammatory effect of blue pigments.

NO was recognized as a mediator and regulator of inflammatory responses and was produced in high amounts by iNOS in activated inflammatory cells [47]. Blue pigments was found to significantly inhibit LPS-induced NO production in a concentration-dependent manner. The mRNA expression of iNOS was also decreased by blue pigments, confirming the suppressive effect of blue pigments on the NO production. The present work also showed that blue pigments inhibited the expression of iNOS mRNA in LPS-stimulated RAW 264.7 macrophage cells. This blocking effect of blue pigments on LPS-induced iNOS expression might have resulted from the transcriptional inhibition of iNOS gene.

The mechanism of various antiinflammatory drug actions was at least shared by the inhibition of prostaglandin synthesis, which was mediated by cycloxygenase (COX) [48]. Of the two isoforms of COX, COX-1 has been suggested to provide a physiologic level of PGs for normal platelet, stomach and kidney function. Moreover, COX-2 has been found to be highly induced at inflammatory sites in animals as well as patients with inflammatory diseases [49], [50]. PGE_2_ was considered one of the strongest inflammatory mediators in inflammatory response. It was transformed from arachidonic acid via the COX-2 catalytic reaction. COX-2 also could be affected directly at its enzymatic activity by NO and iNOS [51]. The results showed that blue pigments significantly reduced PGE_2_ production, and inhibited the COX-2 mRNA and protein expression respectively, in a concentration-dependent manner. It suggested that the anti-inflammatory effect of blue pigments might be attributed to its inhibitive effect on PGE_2_ production through blocking COX-2 gene and protein expression.

In inflammation progress, a series of cytokines and mediators contributed to evoking and regression of inflammation. TNF-α and IL-6 were the critical cytokines involved in inflammation and inhibition of them were regarded as a treatment strategy on inflammation-related diseases [52], [53], [54]. So we chose TNF-α and IL-6 as a parameter to investigate the anti-inflammatory effect of blue pigments. In the present study, we found that TNF-α and IL-6 release and mRNA expressions, which were highly stimulated by LPS, were inhibited by blue pigments. In particular, inhibitions of IL-6 production and mRNA expression were more enhanced than those of TNF-α by blue pigments, which presented the potential of blue pigments to treat typical inflammation -related disorders. For other cytokines involved in the inflammatory process, blue pigments also inhibited their expressions, such as, G-CSF, sICAM-1, IL-1α, , IL-1ra, KC, JE, MIP-1α, MIP-1β, RANTES.

It has been shown that NF-κB activation was a critical factor to expression of various proinflammatory enzymes and cytokines, and iNOS, COX-2, TNF-α, IL-1β and IL-6 in macrophages in response to LPS [14], [15]. NF-κB was composed mainly of two proteins: p50 and p65. In resting cells, the NF-κB heterodimer was held in the cytosol through interaction with IκB inhibitory proteins [55]. NF-κB activation resulted from the phosphorylation and proteasome- mediated degradation of inhibitory IκB proteins, moreover, this was followed by the nuclear translocation and DNA binding of NF-κB [56], [57] where the transcription of target gene was induced. Therefore, we examined NF-κB-DNA binding activity to confirm that the inhibitions of the expressions of iNOS, COX-2, TNF-α and IL-6 are influenced by the NF-κB signaling pathway. Our results indicated that the nuclear translocations of p65 and p50 proteins were inhibited in a concentration-dependent manner by blue pigments and these results corresponded with its inhibition of the expressions of iNOS, COX-2, TNF-α and IL-6. In the cytoplasm, NF-κB is bound to tightly control by its inhibitory subunit, IκB. In the present study, we also found that the translocation of activated NF-κB to the nucleus was inhibited in a concentration-dependent manner by blue pigments, and that the degradation and phosphorylation of IκB-α were also inhibited by blue pigments. These findings indicate that blue pigments may inhibit NF-κB activation by suppressing the phosphorylation of IκB-α and the translocations of the p50 and p65 subunits of NF-κB from the cytosol to the nucleus in LPS-induced RAW 264.7 cells. IKK-α and IKK-β (known as the IκB kinases) are responsible for phosphorylating IκBs [58]. In the present study, we observed that blue pigments inhibited the activation of IKK-α and IKK-β. Thus, we suggest that the inhibition of IKK-α and IKK-β by blue pigments underlies its inhibition of NF-κB activation. Blue pigments (60, 120 mg/kg) showed significant inhibitory effects on carrageenan-induced paw edema in mice, compared with the vehicle control group. Furthermore, blue pigments significantly reduced the plasma TNF-α and IL-6 levels in a dose-dependent manner in the LPS-stimulated animals. Although NF-κB is the major regulator of pro-inflammatory signaling in macrophages, other transcription factors activated by PLS, such as activating protein-1(AP-1), cAMP response element-binding (CREB), The nuclear factor interleukin-6 (NF-IL6) may affect the production of inflammatory mediators[59], [60], [61]. The reason of the little discrepancies in the distinct potency of genipin-derived blue pigment against the LPS-induced activation of NF-κB and the gene expression of some inflammatory mediator might be associated with lack of inhibition of these transcriptional factors (AP-1, CREB, NF-IL6).

In conclusion, although the blue pigments have been used as value-added colorants for foods about 20 years in East Asia, its biological activity has been first explored. The current study demonstrated that blue pigments did not only inhibit iNOS and COX-2 gene expression induced by LPS as well as the subsequent production of NO and PGE_2_, but reduced the production of cytokines (TNF-α, IL-6) induced by LPS in RAW 264.7 macrophages by the inhibition of signaling cascades leading to the activation of NF-κB. Therefore, the results of our studies will provide strong scientific evidence for blue pigments to be developed as a new health-enhancing nutritional food for the prevention and treatment of chronic inflammatory diseases, which also advance our knowledge in molecular nutrition by elucidating pathways for the functional food at a molecular level.

## Supporting Information

Figure S1 **The effect of blue pigments on cell viability in RAW 264.7 cells.** RAW 264.7 cells were treated with the indicated concentrations of blue pigments and then incubated for 24 h. Cell viabilities were assessed using MTT assay. Cell viability was not significantly altered by blue pigments at up to 200 µM. These results suggest that concentrations of blue pigments below 200 µM are not toxic to RAW 264.7 cells.
(TIF)

Figure S2 **Optical density analysis of P-IκB-α (A), IκB-α (B), IKK-α (C) and IKK-β (D).** Each column represents mean ± SD of 4 samples measured by quantitative western blot analysis and normalized by that of β-actin. Measurement was obtained with the Image-Pro Plus version 6.0. * *P*<0.05, ** *P*<0.01 compared with LPS treated cells alone.
(TIF)

Table S1 **Optical density analysis of cytokine protein array.**
(DOC)

## References

[1] Fujikama S, Fukui Y, Koga K, Kumada J (1987). Brilliant skyblue pigment formation from Gardenia fruits.. Journal of Fermentation Technology.

[2] Park JE, Lee JY, Kim HG, Hahn TR, Paik YS (2002). Isolation and characterization of water-soluble intermediates of blue pigments transformed from geniposide of Gardenia jasminoides.. J Agric Food Chem.

[3] Paik Y, Lee C, Cho M, Hahn T (2001). Physical stability of the blue pigments formed from geniposide of gardenia fruits: effects of pH, temperature, and light.. J Agric Food Chem.

[4] Baggiolini M (1998). Chemokines and leukocyte traffic.. Nature.

[5] Palmer RM, Ashton DS, Moncada S (1988). Vascular endothelial cells synthesize nitric oxide from L-arginine.. Nature.

[6] Lowenstein CJ, Hill SL, Lafond-Walker A, Wu J, Allen G (1996). Nitric oxide inhibits viral replication in murine myocarditis.. J Clin Invest.

[7] Hibbs JB, Taintor RR, Vavrin Z (1987). Macrophage cytotoxicity: role for L-arginine deiminase and imino nitrogen oxidation to nitrite.. Science.

[8] Grahames CB, Michel AD, Chessell IP, Humphrey PP (1999). Pharmacological characterization of ATP- and LPS-induced IL-1beta release in human monocytes.. Br J Pharmacol.

[9] Mehta VB, Hart J, Wewers MD (2001). ATP-stimulated release of interleukin (IL)-1beta and IL-18 requires priming by lipopolysaccharide and is independent of caspase-1 cleavage.. J Biol Chem.

[10] Van Snick J (1990). Interleukin-6: an overview.. Annu Rev Immunol.

[11] Marletta MA (1993). Nitric oxide synthase structure and mechanism.. J Biol Chem.

[12] Kleinert H, Pautz A, Linker K, Schwarz PM (2004). Regulation of the expression of inducible nitric oxide synthase.. Eur J Pharmacol.

[13] Harris SG, Padilla J, Koumas L, Ray D, Phipps RP (2002). Prostaglandins as modulators of immunity.. Trends Immunol.

[14] Lawrence T, Gilroy DW, Colville-Nash PR, Willoughby DA (2001). Possible new role for NF-kappaB in the resolution of inflammation.. Nat Med.

[15] Baldwin AS (1996). The NF-kappa B and I kappa B proteins: new discoveries and insights.. Annu Rev Immunol.

[16] Baeuerle PA, Baltimore D (1996). NF-kappa B: ten years after.. Cell.

[17] Surh YJ, Chun KS, Cha HH, Han SS, Keum YS (2001). Molecular mechanisms underlying chemopreventive activities of anti-inflammatory phytochemicals: down-regulation of COX-2 and iNOS through suppression of NF-kappa B activation.. Mutat Res.

[18] Lappas M, Permezel M, Georgiou HM, Rice GE (2002). Nuclear factor kappa B regulation of proinflammatory cytokines in human gestational tissues in vitro.. Biol Reprod.

[19] Makarov SS (2000). NF-kappaB as a therapeutic target in chronic inflammation: recent advances.. Mol Med Today.

[20] Renard P, Raes M (1999). The proinflammatory transcription factor NFkappaB: a potential target for novel therapeutical strategies.. Cell Biol Toxicol.

[21] Cho JY, Kim PS, Park J, Yoo ES, Baik KU (2000). Inhibitor of tumor necrosis factor-alpha production in lipopolysaccharide-stimulated RAW264.7 cells from Amorpha fruticosa.. J Ethnopharmacol.

[22] Hinz B, Brune K, Pahl A (2000). Nitric oxide inhibits inducible nitric oxide synthase mRNA expression in RAW 264.7 macrophages.. Biochem Biophys Res Commun.

[23] Hinz B, Brune K, Pahl A (2000). Prostaglandin E(2) upregulates cyclooxygenase-2 expression in lipopolysaccharide-stimulated RAW 264.7 macrophages.. Biochem Biophys Res Commun.

[24] Seo WG, Pae HO, Oh GS, Kim NY, Kwon TO (2001). The aqueous extract of Rhodiola sachalinensis root enhances the expression of inducible nitric oxide synthase gene in RAW264.7 macrophages.. J Ethnopharmacol.

[25] Suh N, Honda T, Finlay HJ, Barchowsky A, Williams C (1998). Novel triterpenoids suppress inducible nitric oxide synthase (iNOS) and inducible cyclooxygenase (COX-2) in mouse macrophages.. Cancer Res.

[26] Fujikama S, Fukui Y, Koga K (1987). Structure of genipocyanin G1, a spontaneous reaction product between genipin and glycine.. Tetrahedron Letters.

[27] Versteeg HH, Nijhuis E, van den Brink GR, Evertzen M, Pynaert GN (2000). A new phosphospecific cell-based ELISA for p42/p44 mitogen-activated protein kinase (MAPK), p38 MAPK, protein kinase B and cAMP-response-element-binding protein.. Biochem J.

[28] Wang QS, Cui YL, Wang YF, Chi W (2011). Effects of compounds from Bi-Qi Capsule on the expression of inflammatory mediators in lipopolysaccharide-stimulated RAW 264.7 macrophages.. J Ethnopharmacol.

[29] Raspotnig G, Fauler G, Jantscher A, Windischhofer W, Schachl K (1999). Colorimetric determination of cell numbers by Janus green staining.. Anal Biochem.

[30] Kang HG, Jenabi JM, Zhang J, Keshelava N, Shimada H (2007). E-cadherin cell-cell adhesion in ewing tumor cells mediates suppression of anoikis through activation of the ErbB4 tyrosine kinase.. Cancer Res.

[31] Marcondes AM, Mhyre AJ, Stirewalt DL, Kim SH, Dinarello CA (2008). Dysregulation of IL-32 in myelodysplastic syndrome and chronic myelomonocytic leukemia modulates apoptosis and impairs NK function.. Proc Natl Acad Sci U S A.

[32] Feron M, Guevel L, Rouger K, Dubreil L, Arnaud MC (2009). PTEN contributes to profound PI3K/Akt signaling pathway deregulation in dystrophin-deficient dog muscle.. Am J Pathol.

[33] Livak KJ, Schmittgen TD (2001). Analysis of relative gene expression data using real-time quantitative PCR and the 2(−Delta Delta C(T)) Method.. Methods.

[34] De Gois S, Schafer MK, Defamie N, Chen C, Ricci A (2005). Homeostatic scaling of vesicular glutamate and GABA transporter expression in rat neocortical circuits.. J Neurosci.

[35] Shin EM, Zhou HY, Guo LY, Kim JA, Lee SH (2008). Anti-inflammatory effects of glycyrol isolated from Glycyrrhiza uralensis in LPS-stimulated RAW264.7 macrophages.. Int Immunopharmacol.

[36] Fukuzawa M, Satoh J, Sagara M, Muto G, Muto Y (1997). Angiotensin converting enzyme inhibitors suppress production of tumor necrosis factor-alpha in vitro and in vivo.. Immunopharmacology.

[37] Tanaka M, Yamazaki M, Chiba K (2009). Neuroprotective action of genipin on tunicamycin induced cytotoxicity in neuro2a cells.. Biol Pharm Bull.

[38] Koo HJ, Song YS, Kim HJ, Lee YH, Hong SM (2004). Antiinflammatory effects of genipin, an active principle of gardenia.. Eur J Pharmacol.

[39] Takeuchi S, Goto T, Mikami K, Miura K, Ohshima S (2005). Genipin prevents fulminant hepatic failure resulting in reduction of lethality through the suppression of TNF-alpha production.. Hepatol Res.

[40] Chang WH, Chang Y, Lai PH, Sung HW (2003). A genipin-crosslinked gelatin membrane as wound-dressing material: in vitro and in vivo studies.. J Biomater Sci Polym Ed.

[41] Sung HW, Huang DM, Chang WH, Huang LL, Tsai CC (1999). Gelatin-derived bioadhesives for closing skin wounds: an in vivo study.. J Biomater Sci Polym Ed.

[42] Liu BS, Yao CH, Chen YS, Hsu SH (2003). In vitro evaluation of degradation and cytotoxicity of a novel composite as a bone substitute.. J Biomed Mater Res A.

[43] Tsai CC, Chang Y, Sung HW, Hsu JC, Chen CN (2001). Effects of heparin immobilization on the surface characteristics of a biological tissue fixed with a naturally occurring crosslinking agent (genipin): an in vitro study.. Biomaterials.

[44] Levinton-Shamuilov G, Cohen Y, Azoury M, Chaikovsky A, Almog J (2005). Genipin, a novel fingerprint reagent with colorimetric and fluorogenic activity, part II: optimization, scope and limitations.. J Forensic Sci.

[45] Zhang CH (2006). Food Colorants Data Book.

[46] Rafi MM, Shafaie Y (2007). Dietary lutein modulates inducible nitric oxide synthase (iNOS) gene and protein expression in mouse macrophage cells (RAW 264.7).. Mol Nutr Food Res.

[47] Korhonen R, Lahti A, Kankaanranta H, Moilanen E (2005). Nitric oxide production and signaling in inflammation.. Curr Drug Targets Inflamm Allergy.

[48] Vane JR (1971). Inhibition of prostaglandin synthesis as a mechanism of action for aspirin-like drugs.. Nat New Biol.

[49] Masferrer JL, Zweifel BS, Manning PT, Hauser SD, Leahy KM (1994). Selective inhibition of inducible cyclooxygenase 2 in vivo is antiinflammatory and nonulcerogenic.. Proc Natl Acad Sci U S A.

[50] Seibert K, Zhang Y, Leahy K, Hauser S, Masferrer J (1994). Pharmacological and biochemical demonstration of the role of cyclooxygenase 2 in inflammation and pain.. Proc Natl Acad Sci U S A.

[51] Moncada S, Palmer RM, Higgs EA (1991). Nitric oxide: physiology, pathophysiology, and pharmacology.. Pharmacol Rev.

[52] Locksley RM, Killeen N, Lenardo MJ (2001). The TNF and TNF receptor superfamilies: integrating mammalian biology.. Cell.

[53] Burger D, Dayer JM, Palmer G, Gabay C (2006). Is IL-1 a good therapeutic target in the treatment of arthritis?. Best Pract Res Clin Rheumatol.

[54] Rose-John S, Waetzig GH, Scheller J, Grotzinger J, Seegert D (2007). The IL-6/sIL-6R complex as a novel target for therapeutic approaches.. Expert Opin Ther Targets.

[55] Baeuerle PA, Henkel T (1994). Function and activation of NF-kappa B in the immune system.. Annu Rev Immunol.

[56] Brown K, Park S, Kanno T, Franzoso G, Siebenlist U (1993). Mutual regulation of the transcriptional activator NF-kappa B and its inhibitor, I kappa B-alpha.. Proc Natl Acad Sci U S A.

[57] Rodriguez MS, Thompson J, Hay RT, Dargemont C (1999). Nuclear retention of IkappaBalpha protects it from signal-induced degradation and inhibits nuclear factor kappaB transcriptional activation.. J Biol Chem.

[58] May MJ, Ghosh S (1999). IkappaB kinases: kinsmen with different crafts.. Science.

[59] Jeon YJ, Han SH, Lee YW, Lee M, Yang KH (2000). Dexamethasone inhibits IL-1 beta gene expression in LPS-stimulated RAW 264.7 cells by blocking NF-kappa B/Rel and AP-1 activation.. Immunopharmacology.

[60] Avni D, Ernst O, Philosoph A, Zor T (2010). Role of CREB in modulation of TNFalpha and IL-10 expression in LPS-stimulated RAW264.7 macrophages.. Mol Immunol.

[61] Godambe SA, Chaplin DD, Takova T, Bellone CJ (1994). Upstream NFIL-6-like site located within a DNase I hypersensitivity region mediates LPS-induced transcription of the murine interleukin-1 beta gene.. J Immunol.

