# Supplementary figures and images for: Dietary Blue Pigments Derived from Genipin, Attenuate Inflammation by Inhibiting LPS-Induced iNOS and COX-2 Expression via the NF-κB Inactivation
Source: PLoS One. 2012 Mar 30;7(3):e34122. doi: 10.1371/journal.pone.0034122 (PMC3316609; doi:10.1371/journal.pone.0034122)

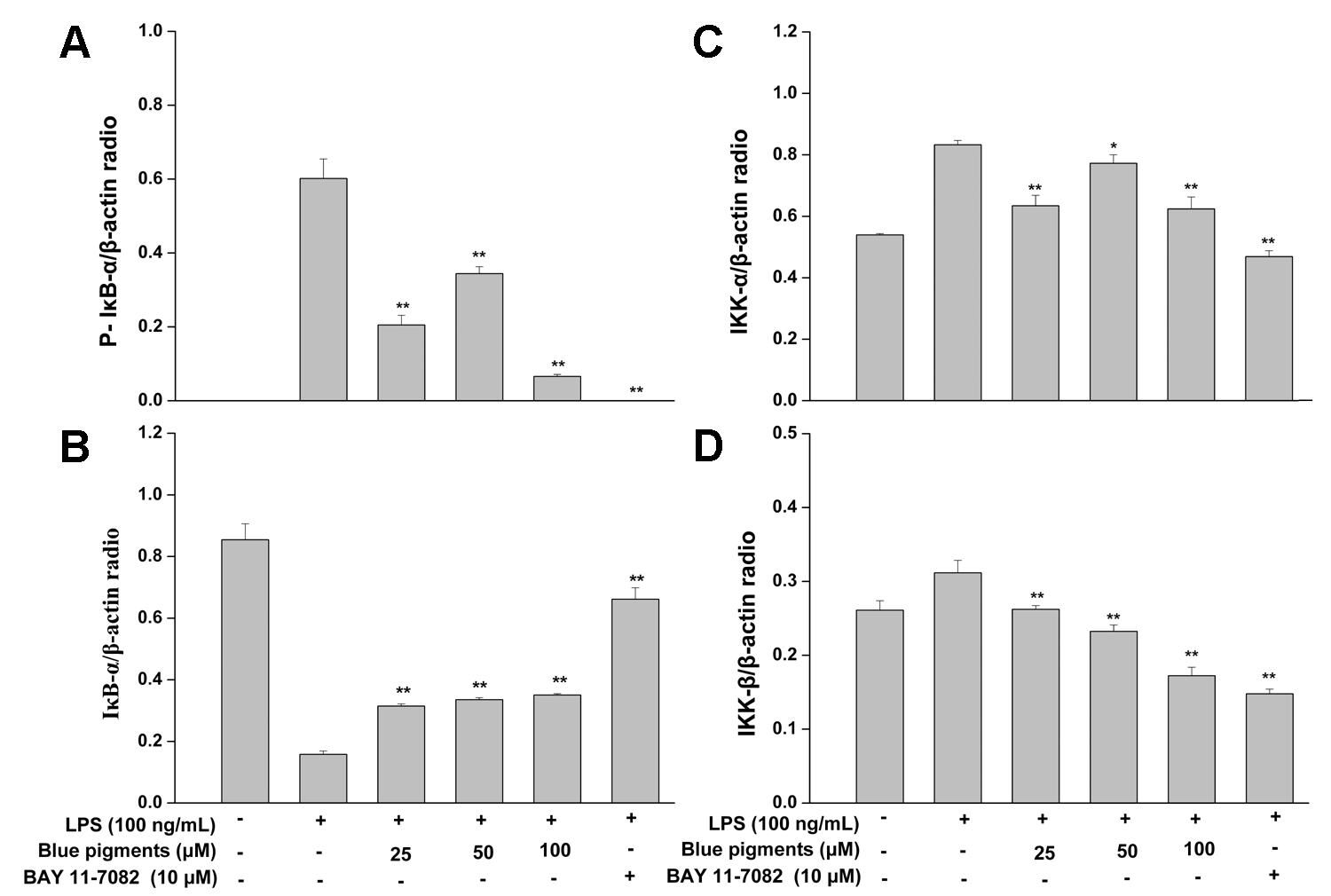

Supplement: Figure S2 — Optical density analysis of P-IκB-α (A), IκB-α (B), IKK-α (C) and IKK-β (D). Each column represents mean ± SD of 4 samples measured by quantitative western blot analysis and normalized by that of β-actin. Measurement was obtained with the Image-Pro Plus version 6.0. * P<0.05, ** P<0.01 compared with LPS treated cells alone. (TIF) [file pone.0034122.s002.tif]
