# Supplementary material for: Dietary Blue Pigments Derived from Genipin, Attenuate Inflammation by Inhibiting LPS-Induced iNOS and COX-2 Expression via the NF-κB Inactivation
Source: PLoS One. 2012 Mar 30;7(3):e34122. doi: 10.1371/journal.pone.0034122 (PMC3316609; doi:10.1371/journal.pone.0034122)
Supplement: Table S1 — Optical density analysis of cytokine protein array. (DOC) [file pone.0034122.s003.doc]

**Table S1. Optical density analysis of cytokine protein array.**

|  | G-CSF | sICAM-1 | IL-1α | IL-1β | IL-1ra | KC | JE | MIP-1α | MIP-1β | MIP-2 | RANTES | TNF-α |
| --- | --- | --- | --- | --- | --- | --- | --- | --- | --- | --- | --- | --- |
| LPS | 0.0243 | 0.2371 | 0.4714 | 0.0188 | 0.9000 | 0.0849 | 0.4900 | 0.5780 | 0.0714 | 0.9634 | 0.9503 | 0.0121 |
|  | 0.0269 | 0.2228 | 0.4829 | 0.0204 | 0.8428 | 0.0957 | 0.4662 | 0.5507 | 0.0831 | 1.0676 | 1.0769 | 0.0159 |
| BP | 0.0077 | 0.1941 | 0.3182 | 0.0166 | 0.6035 | 0.0589 | 0.3097 | 0.4909 | 0.0358 | 0.7152 | 0.4810 | 0.0131 |
|  | 0.0102 | 0.1970 | 0.3301 | 0.0166 | 0.6452 | 0.0656 | 0.3197 | 0.5495 | 0.0405 | 0.8002 | 0.5743 | 0.0127 |
| ***P*** | * | ** | ** | # | ** | * | ** | # | * | # | * | # |

BP: blue pigments

Data represent means ± S.D. values. * *P* < 0.05, ** *P* < 0.01 compared with LPS treated cells alone, # *P* > 0.05 compared with LPS treated cells alone.
